# Identification of cancer subtypes from single-cell RNA-seq data using a consensus clustering method

**DOI:** 10.1186/s12920-018-0433-z

**Published:** 2018-12-31

**Authors:** Yanglan Gan, Ning Li, Guobing Zou, Yongchang Xin, Jihong Guan

**Affiliations:** 1 School of Computer Science and Technology, Donghua University, Shanghai, China; 2 School of Computer Engineering and Science, Shanghai University, Shanghai, China; 3 Department of Computer Science and Technology, Tongji University, Shanghai, China

**Keywords:** Consensus clustering, Intratumoral heterogeneity, Cancer subtypes, Single-cell sequencing

## Abstract

**Background:**

Human cancers are complex ecosystems composed of cells with distinct molecular signatures. Such intratumoral heterogeneity poses a major challenge to cancer diagnosis and treatment. Recent advancements of single-cell techniques such as scRNA-seq have brought unprecedented insights into cellular heterogeneity. Subsequently, a challenging computational problem is to cluster high dimensional noisy datasets with substantially fewer cells than the number of genes.

**Methods:**

In this paper, we introduced a consensus clustering framework conCluster, for cancer subtype identification from single-cell RNA-seq data. Using an ensemble strategy, conCluster fuses multiple basic partitions to consensus clusters.

**Results:**

Applied to real cancer scRNA-seq datasets, conCluster can more accurately detect cancer subtypes than the widely used scRNA-seq clustering methods. Further, we conducted co-expression network analysis for the identified melanoma subtypes.

**Conclusions:**

Our analysis demonstrates that these subtypes exhibit distinct gene co-expression networks and significant gene sets with different functional enrichment.

## Background

Characterization of intratumoral heterogeneity is critical to precision cancer therapy, as diverse cell populations usually enable relapse and resistance to treatment [1]. Conventional bulk RNA-seq technology reveals the average gene expression of a collection of cells, and subsequently many methods have been developed to inference tumor evolution using data from bulk-sequencing of tumor samples [2, 3]. These approaches require deconvolution of the mixed signals of the underlying tumor subpopulations, which are often ambiguous [4].

Recently, single-cell RNA-Seq (scRNA-seq) quantifies the expression of diverse cellular populations and enables researchers to analyze the difference among cells [5–7]. A full characterization of the transcriptional landscape of individual cell holds enormous potential for detection of clinically important tumor subpopulations, understanding of tumor heterogeneity and further clinical applications [8, 9]. Clustering of single-cell expression data provides an intuitive way for identification of cell types from a mass of heterogeneous cells, which can be used in diverse downstream expression analysis [10–12].

Due to noise, high dimensionality and data heterogeneity, newly produced scRNA-seq data pose a grand challenge for traditional clustering algorithms, such as K-means, hierarchical clustering, and spectral clustering [13]. One feasible strategy is to first reduce the high dimensional data into a lower dimensional subspace and apply traditional clustering to the dimension-reduced data. The widely used dimension reduction methods include principal component analysis (PCA) [14] or t-Distributed Stochastic Neighbor Embedding algorithm (t-SNE) [15]. Meanwhile, a number of methods which specifically designed for scRNA-seq analysis have been introduced, including Seurat [16], CIDR [17], SNN-cliq [18], SINCERA [19] and SC3 [20]. These advanced methods have greatly improved the capability of scRNA-seq data analysis. However, as clustering methods are mostly sensitive to noise and initial parameters, how to accurately cluster scRNA-seq data across different environments and revealing biological insights is still a substantial challenge [21].

Here, we proposed a consensus clustering model, conCluster, for cancer subtype identification from single-cell RNA-seq data. Specifically, conCluster first obtains a set of basic partitions using tSNE+K-means clustering with different initial parameters, and then fuses these different partitions into consensus clusters. Our conCluster method can also be easily extended to ensemble the clustering results of different clustering methods. We applied conCluster to real cancer scRNA-seq datasets, and further constructed the co-expression networks for the identified cancer subtypes to analyze their difference. The experimental results demonstrate the effectiveness and robustness of conCluster compared with five widely used clustering methods.

## Methods

### Overview of the conCluster model

**Fig. 1:**
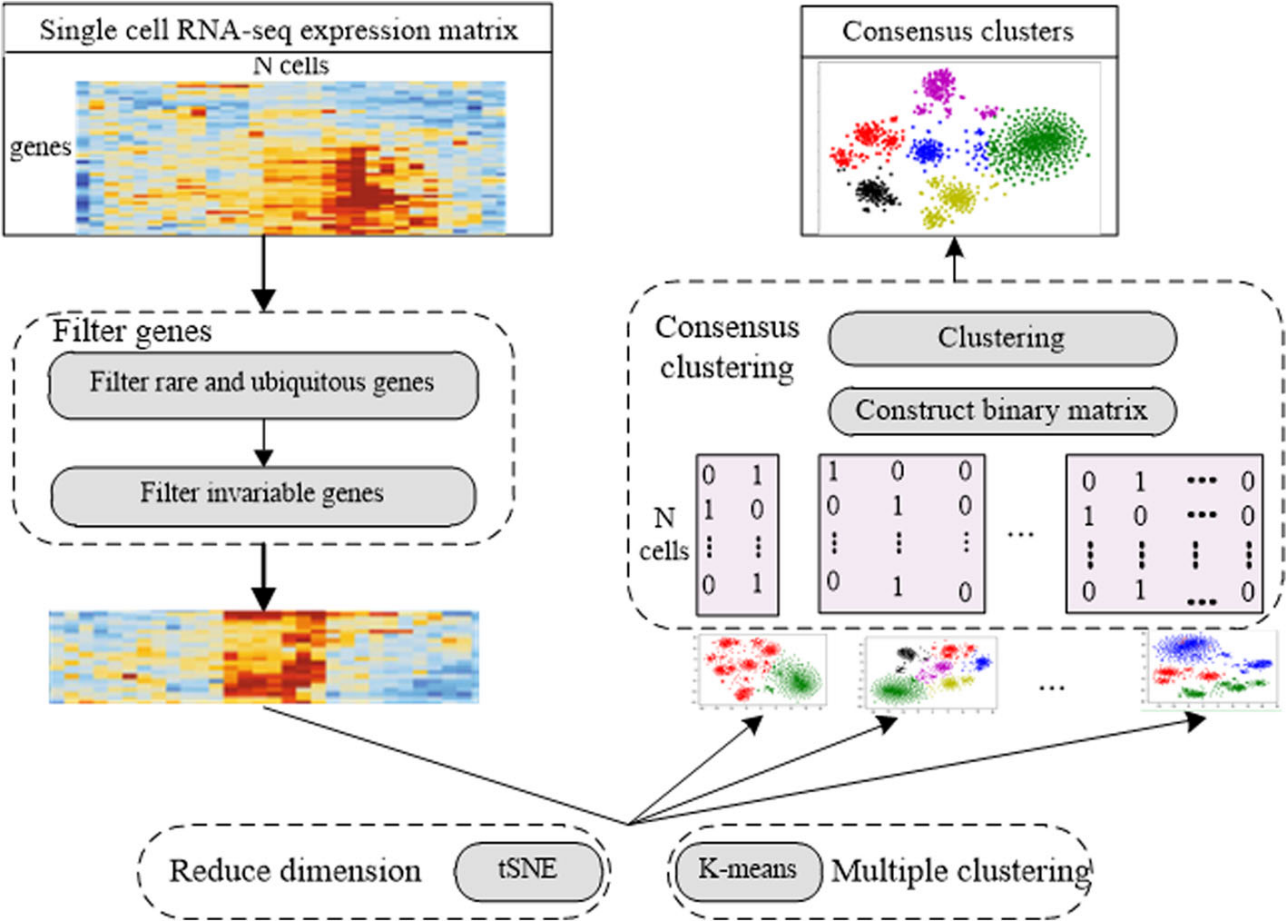
The schematic workflow of conCluster

To identify subtypes from a collection of cancer cells, we developed conCluster to ensemble multiple clustering results. Let *E*_*N*∗*G*_ denotes a single-cell gene expression matrix, in which rows correspond to different cells and columns correspond to genes. Each element of *E*_*ij*_ corresponds to the expression of gene *j* in the *i*th cell. Our conCluster takes the expression matrix *E* as input, through four steps, finally partition the *N* cells into *K* clusters, represented as *C*={*C*_*k*_|*k*=1,2,⋯,*K*}. Figure 1 shows the overview of the proposed conCluster model. In the following, we will elaborate each step in detail.

#### Step1 Filter genes

To focus on the intrinsic transcriptomic signatures of these tumor cells, we filtered out rare and ubiquitous genes and identified the most variable genes across the single-cell dataset. Firstly, as the rare and ubiquitous genes are usually not useful for clustering, we filtered out genes that are either expressed in less than *r*% of cells (rare genes) or expressed in at least (100-*r*)% of cells (ubiquitous genes). As in the previous study [22], *r* is set as 6. Next, we identified the gene set that was the most *v*% variable across these single-cells, by controlling the relationship between mean expression and variability.

#### Step 2 Reduce dimension using t-SNE

To further reduce the dimensionality, we adopted the widely used t-SNE to reduce the high dimensional data into a lower dimensional subspace. Detailedly, perplexity is an important parameter of t-SNE, which is used as a smooth measure of the effective number of neighbors. Previous studies indicate that performance of t-SNE is fairly robust with changes in the perplexity between 5 and 50. Here, we set perplexity as 30 and used t-SNE to reduce the filtered scRNA expression data into two dimensions.

#### Step3 Partition cells in multiple ways

Based on the transformed two-dimensional data matrices, we performed K-means clustering with different initial parameters *T* times to obtain different basic partitions for these single cells. In this step, we can also utilize other basic clustering methods. For each individual clustering result, we derived a binary matrix *B*_*N*∗*Kt*_, which was constructed based the corresponding cluster labels of *N* cells, where *Kt* (*t*=1,2,⋯,*T*) is the cluster number in the *t*th basic partition. For each row of *B*_*N*∗*Kt*_, only one element is 1, others are 0.

#### Step4 Consensus clustering

After gaining the *T* different partitions, we concatenated all those binary matrices into a larger binary matrix *B*={*B*_*N*∗*Kt*_|*t*=1,2,⋯,*T*}. Furthermore, we performed K-means clustering based on the merged binary matrix. Here, Calinski-Harabaz Index [22] is utilized to decide the number of clusters. Then we fused the results of each individual clustering result into a consensus one [23].

### Evaluation Metrics

When cell labels are available in the dataset, we adopted the adjusted rand index (ARI) to measure the accuracy of clustering [24]. For a set of *N* cells and two different partitions of these cells, the overlap between the two partitions can be summarized in a contingency table, in which each entry denotes the number of objects in common between the two partitions. The ARI is then calculated as:

1 $$ ARI=\frac{\sum\limits_{ij}{{n_{ij}} \choose {2}} -\left[\sum\limits_{i} {{a_{i}} \choose {2}} \sum\limits_{j} {{b_{i}} \choose {2}} \right]/{{n} \choose {2}}}{\left[\sum\limits_{i} {{a_{i}} \choose {2}} +\sum\limits_{j} {{b_{i}} \choose {2}} \right]/2-\left[\sum\limits_{i} {{a_{i}} \choose {2}} \sum\limits_{j} {{b_{i}} \choose {2}} \right]/{{n} \choose {2}}}  $$

where (.) denotes a binomial coefficient, *n*_*ij*_ is the element from the contingency table, *a*_*i*_ is the sum of the *i*th row of the contingency table, *b*_*j*_ is the sum of the *j*th column of the contingency table.

### Datasets

Single-cell expression data from two recent scRNA-seq studies were selected from the data repository NCBI Gene Expression Omnibus (GSE72056 [25], GSE73727 [26]).

As they contained the cell types in the original publications, it can be used to further validate the clustering results of different methods. In these studies, cell types were determined through a multi-stage process involving additional information such as cell-type molecular signatures. The first dataset contains a collection of cells from human melanoma tumor, consisted of 4645 single cells isolated from 19 patients; and the second dataset is from human pancreatic islet, containing 6 known human islet cell types. To ensure good data quality, samples with a library size less than 10,000 were excluded. Data sets transformed by logTPM were used as inputs of different methods.

## Results

### Performance evaluation on single cell RNA-seq data

To fully evaluate the performance, we compared conCluster with five widely used scRNA-seq data clustering methods, including spectral clustering, tSNE+K-means, SNN-Cliq, CIDR and SC3. Specifically, spectral clustering is an efficient traditional clustering method; tSNE+K-means is K-means clustering combined with the nonlinear dimensionality reduction technique tSNE; SNN-Cliq adopts a shared nearest neighbours approach to calculate similarities between cells and performs single cell clustering using a graph-theoretical model; CIDR uses an imputation approach to alleviate the impact of dropouts in scRNA-seq data in a principled manner; and SC3 transforms a cell-to-cell distance matrix from individual K-means clustering to get a consensus partitions. To run the main SC3 method, the parameter ks is required to set. For CIDR, there are two parameters (nPC and nCluster). SNN-cliq relies on four paramters (k, distance r and m). We tried different values of these parameters and selected those values which obtain the highest ARI. Here, we selected two single cell RNA-seq datasets [GSE72056 and GSE73727], as they contained preexisting cluster structures that can be used for validation.

**Fig. 2:**
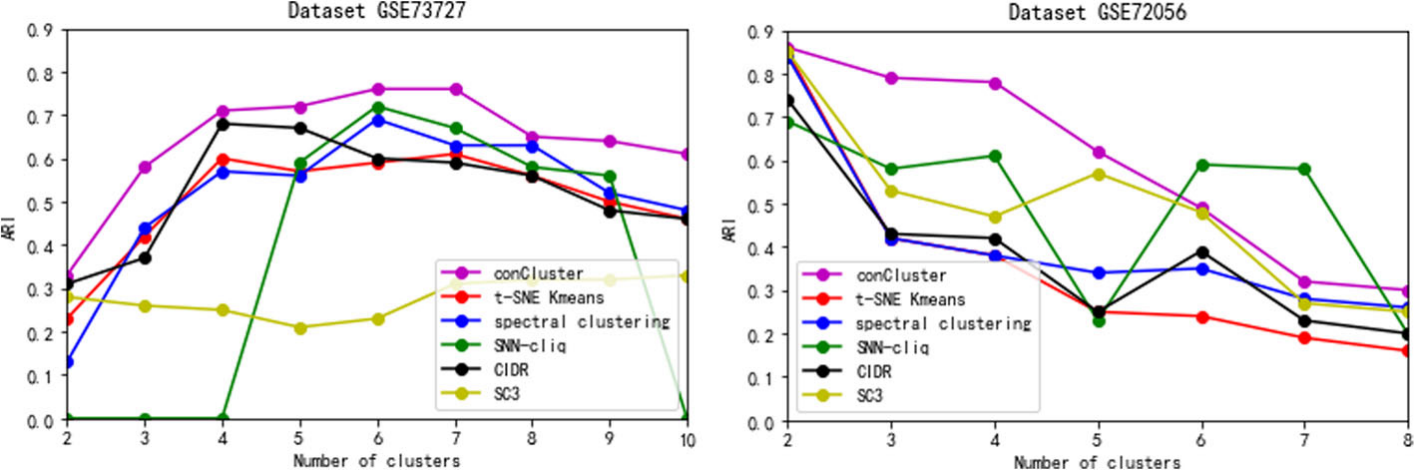
Performance evaluation of conCluster and five widely-used scRNA-data clustering methods. Adjusted Rand Index (ARI) is employed to measure the similarity between inferred and true cluster labels

Figure 2 shows the clustering performance of different algorithms as measured by the adjusted rand index (ARI). For dataset GSE73727 with 6 clusters, these methods achieve better performance when the cluster number is close to 6. For dataset GSE72056 with 2 clusters (malignant and benign tumor), the performance is the best when k equals to 2. Overall, the proposed conCluster identifies the subtypes of these single cells more correctly, as reflected by an adjusted rand index close to 0.9, which is higher than those of the five compared methods for both the datasets. Some methods such as SNN-cliq and SC3 can get comparable performance to conCluster. However, their performance is not stable for different datasets and clusters. The ARI of traditional clustering method such as spectral clustering is relative lower than other methods. Although the performance of tSNE+K-means is not so good and K-means exhibits the stochasticity in the clustering structures due to the random initialization, our conCluster based on multiple tSNE+K-means gains better solutions than other methods. Its performance suggests that the ensemble of multiple partitions of the data helps to merge clusters together in a sensible way.

### Identification of cancer subtypes

**Fig. 3:**
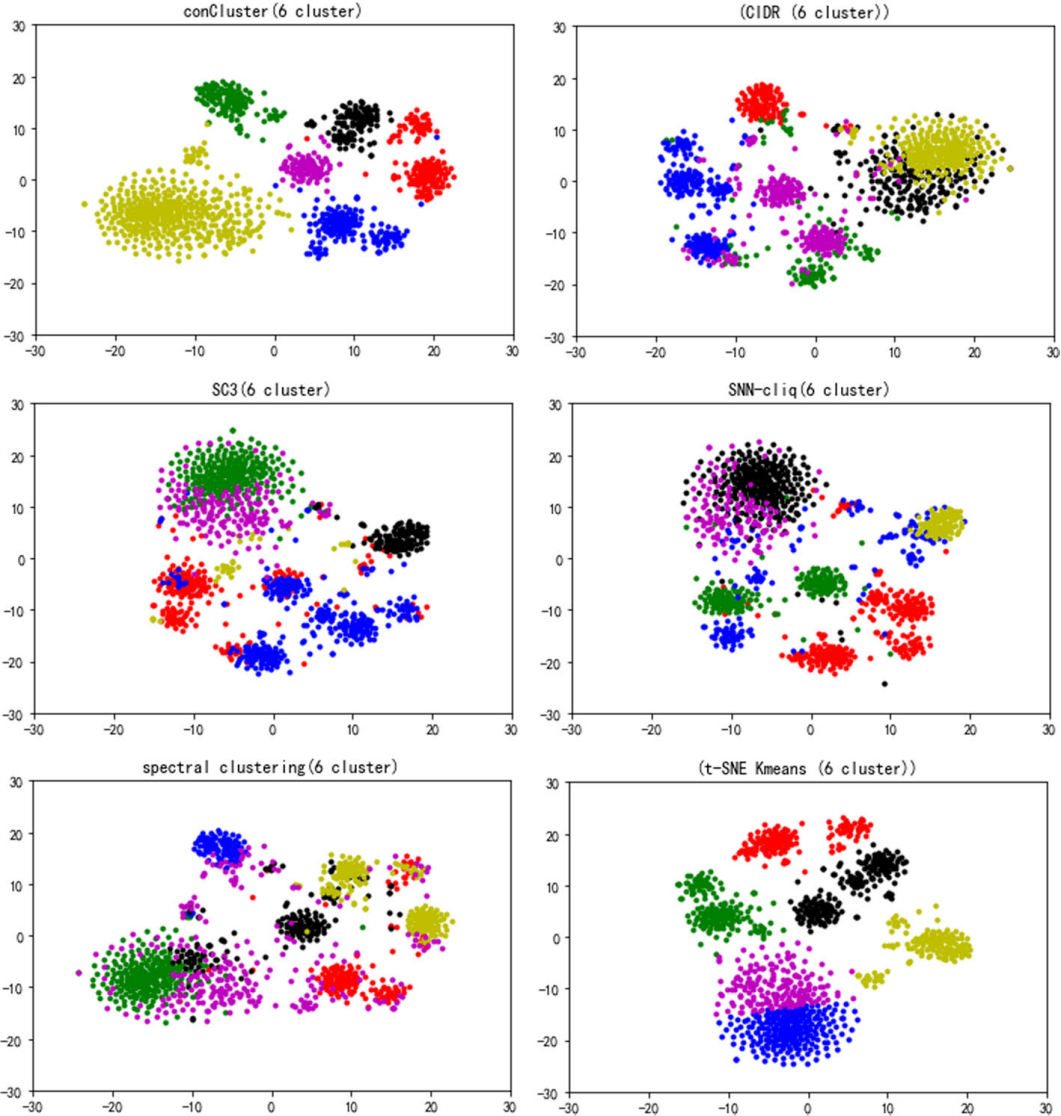
Identification of subtypes from the human melanoma scRNA-seq data set. The different colors denote the clusters output by each algorithm (Clusters numbers k = 6)

Further, we applied conCluster and the five compared algorithms on the malignant melanoma tumor cells in GSE72056. In this dataset, there are 1257 malignant cells after excluding benign tumor cells. Determining the number of clusters is known to be difficult in clustering. As there is not ground truth of the clusters for these malignant cells, we utilized Calinski-Harabaz Index [22] to determine the number of clusters. conCluster managed to identify six clusters in the dataset. As shown in Fig. 3, conCluster displays five more clearly recognizable clusters than the compared methods. SNN-cliq, tSNE+K-means and SC3 also get relatively clear clusters, whereas spectral clustering and CIDR did not perform well in differentiating these clusters.

**Fig. 4:**
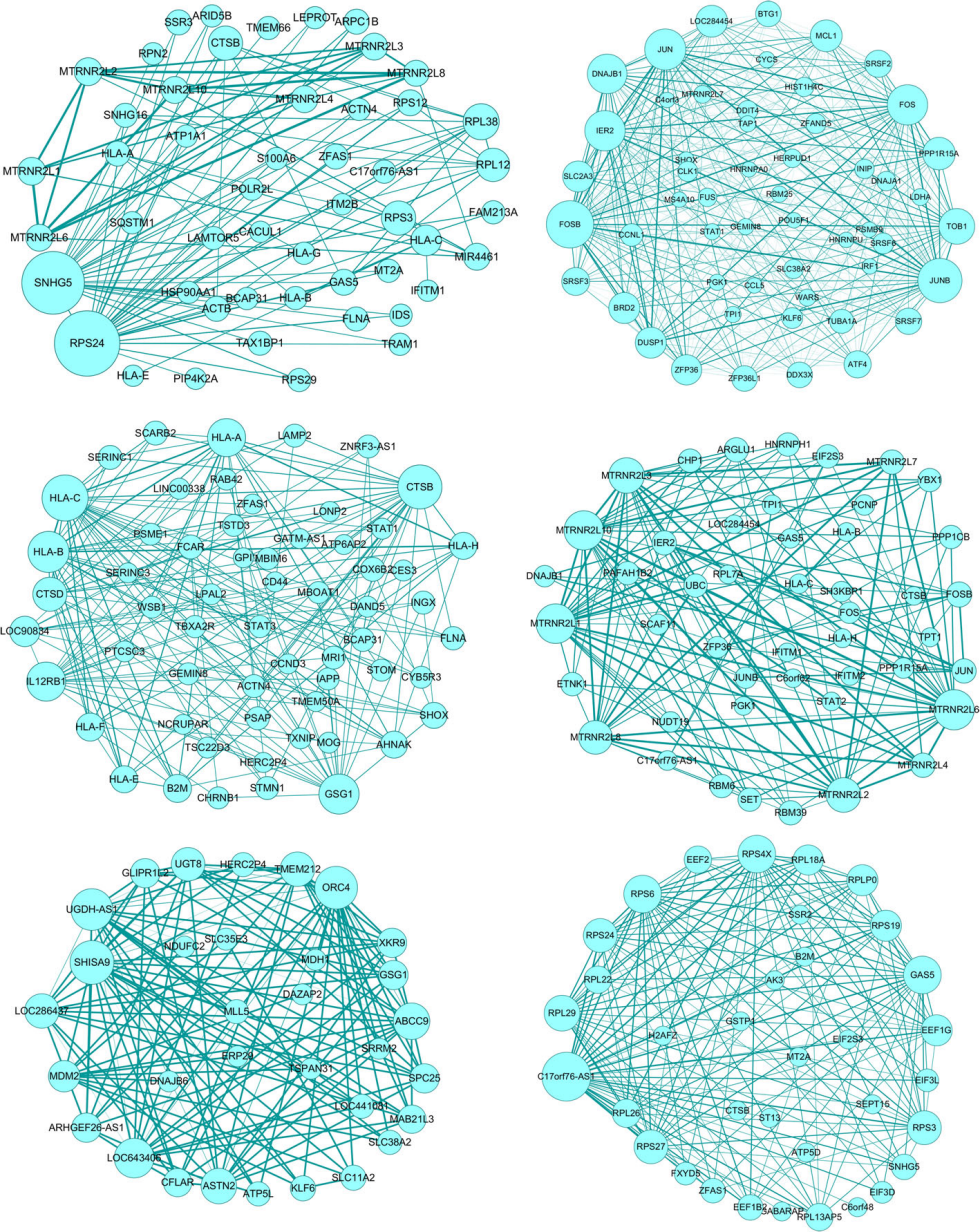
The co-expression networks are visualized for six different subtypes of human malignant melanoma tumor. Node represents gene, Edge weight indicates the statistical significance of co-expression relationship

Next, to identify the regulatory genes of each subtype of the malignant melanoma, we conducted gene co-expression network analysis. A co-expression network identifies which genes have a tendency to show a coordinated expression pattern in specific subtype. This co-expression network can be represented as a gene¨Cgene similarity matrix. Here, we identified genes having significant expression difference among cells by applying a 5% FDR. These genes were used to reconstruct the subtype specific co-expression network and identify a number of modules of high co-expression genes. we utilized WGCNA to construct co-expression modules, which is a widely used tool for co-expression analysis. Figure 4 shows the co-expression network for each melanoma subtype. We noticed that different subtypes include the distinct co-expression gene subsets. These genes with the highest degree of connectivity usually are expected to be drivers which are required for signaling pathways of essential function.

We computed the network degree for each gene in the co-expression networks of different melanoma subtypes, and identify genes with the most connections. To examine the potential functions of those genes, we performed systematic gene ontology enrichment analysis using DAVID tools and summarized the key biological processes and pathways [27]. The most highly connected genes in each network and the corresponding gene ontology enrichment analysis are listed in Table 1. Overall, these modules are significantly enriched for biologically important processes that are relevant to melanoma, including response to light stimulus, antigen processing and regulation of cell death. For example, in subtypes 1, the most connected gene was involved in translation initiation (RPL12, RPL38, RPS24, RPS3); in subtypes 2, the set of most highly connected genes included genes involved in cellular response to stimulus response (FOS, DUSP1,JUN, FOSB); in subtypes 3, genes sets including B2M, HLA-A, HLA-B, are related with antigen processing and presentation.

**Table 1:** Significant genes and GO analysis of the co-expression networks of different melanoma subtypes

	Gene list	Term type & name	*P*-value
Subtype 1	RPS24 SNHG5 RPS3 RPL38	BP: cotranslational protein targeting	6.34E-8
	RPL12 CTSB PAICS HLA-C	BP: translational initiation	2.89E-7
	RPS12 MTRNR2L2 MTRNR2L6	BP: regulation of apoptotic process	9.71E-2
	GAS5 MTRNR2L10 ARPC1B	KEGG: Antigen processing and presentation	8.47E-2
		CC: focal adhesion	1.42E-3
Subtype 2	FOSB JUNB JUN IER2	BP: cellular response	2.28E-6
	DNAJB1 TOB1 PPP1R15A	BP: negative regulation of transcription	7.67E-3
	LOC284454 MCL1 BRD2	BP: regulation of cell death	3.025E-3
	DUSP1 SLC2A3 ZFP36	MF: transcription factor activity	1.65E-4
		KEGG: Osteoclast differentiation	4.96 E-4
Subtype 3	HLA-C CTSB HLA-B GSG1	BP: interferon signaling pathway	2.09E-12
	IL12RB1 HLA-A CTSD	BP: positive regulation of T cell mediated cytotoxicity	2.96E-9
	LOC90834 B2M HLA-F HLA-H	BP:antigen processing	1.38E-6
	AHNAK HLA-E SHOX	BP: immune response	5.22E-11
		KEGG:A ntigen processing and presentation	8.11E-8
Subtype 4	MTRNR2L6 MTRNR2L10	BP: cellular response to hormone stimulus	1.33E-2
	MTRNR2L1 MTRNR2L3	BP: response to mechanical stimulus	1.50E-2
	MTRNR2L2 MTRNR2L8	KEGG: Osteoclast differentiation	1.89E-2
	FOSB IER2 MTRNR2L4	MF:DNA binding	8.34E-3
	MTRNR2L7 ARGLU1	MF: transcription factor activity	6.79E-3
	JUN RBM39 SET		
Subtype 5	SHISA9 ABCC9 ORC4	KEGG: Cell cycle	5.28E-2
	UGDH-AS1 LOC643406 ASTN2	CC: integral component of membrane	7.95E-2
	MDM2 UGT8 LOC286437	CC: synapse	8.59E-2
	TMEM212 XKR9 GLIPR1L2		
	SPC25 ARHGEF26-AS1		
Subtype 6	C17orf76-AS1 RPS4X	BP:translational initiation	1.21E-18
	RPS6 GAS5 RPL29	BP:rRNA processing	7.40E-17
	RPS3 RPS24 RPS27	BP:ribosomal small subunit biogenesis	8.07E-5
	EEF1G RPS19 RPLP0	MF:structural constituent of ribosome	1.76E-15
	RPL18A RPL26 RPL13AP5	KEGG:Ribosome	2.44E-10

## Conclusions

Cancers usually exhibit substantial tumor heterogeneity in virtually all distinguishable phenotypic features, such as cellular morphology, gene expression and metabolism. In order to analyze tumor heterogeneity, it is important to correctly group cell population into different subtypes based on single-cell expression data. Due to the unavoidable biological and technical variations, these scRNA-seq dataseta are noisy and high dimensional, which poses great challenges to the computational methods. In this paper, we proposed, conCluster, an unsupervised consensus clustering method to overcome these limitation and provide robust clustering. Specifically, our conCluster fuses many basic partitions to a consensus one, this procedure may reduce the impact that the performances of individual clustering method tend to affected by noises and different initial parameters. Moreover, data preprocessing steps such as dimensionality reduction is important in scRNA-seq data analysis. The experimental result indicates that the proposed conCluster can more accurately detect cancer subtypes than the compared widely used scRNA-seq clustering methods. The performance improvement of conCluster will be of interest to researchers in the field of scRNA-seq data analysis.

## Abbreviations

CIDR: Clustering through imputation and dimensionality reduction
DAVID: The database for annotation, visualization and integration discovery
PCA: Principal component analysis
SC3: Single-cell consensus clustering
scRNA-seq: Single-cell RNA sequencing
SINCERA: SINgle CEll RNA-seq profiling analysis
SNN-Cliq: Shared nearest neighbor-Cliq
t-SNE: t-distributed stochastic neighbor embedding algorithm
WGCNA: Weighted gene co-expression network analysis
